# Asthma diagnosis and treatment – 1018. Effect of inhaled corticosteroids with long-acting beta2 agonists vs. inhaled corticosteroids alone on asthma control in children: results from national ashtma survey

**DOI:** 10.1186/1939-4551-6-S1-P18

**Published:** 2013-04-23

**Authors:** Richard F Lockey, Neetu Talreja, Dennis K Ledford

**Affiliations:** 1 Division of Allergy Immunology, Department of Medicine, Morsani University of South Florida College of Medicine and James A Haley Veterans Hospital, Tampa, FL, USA

## Background

There is limited evidence for the comparative efficacy of inhaled corticosteroids (ICS) vs. ICS used in combination with a long-acting beta2 agonist (ICS/LABA) to treat asthma in children under 12 years.

## Methods

Data from the National Asthma Survey were analyzed to compare asthma control in children (5 to 11 yrs) using ICS/LABA vs. ICS alone. Both short term (symptoms within last 2 weeks, day and night symptoms in last 30 days and use of systemic glucocorticoids in last 3 months) and long term (asthma attack, emergency department visits, hospitalizations and activity limitations in the prior year) outcomes were compared. Demographics, availability of health insurance, indoor allergen exposure and asthma education were compared between the two groups. Asthma control in an adult population (18 to 44 years) was also assessed for the same parameters.

## Results

69 children using ICS/LABA and 134 using ICS alone were identified. Baseline characteristics were similar in both groups. 67% of children were using ICS/LABA for more than 6 months. There were no differences in short and long term asthma outcomes between the two groups (p>0.05 for all outcome measures). Similar results were obtained with the adult population. (p>0.05 for all outcome measures).

## Conclusions

These data indicate that asthma control in children, using ICS/LABA vs ICS alone, was not different than asthma control using similar medications in the adult population. LABA tolerance or the use of ICS/LABA in subjects with more severe asthma could explain these findings.

